# What are we paying for? A cost-effectiveness analysis of patented denosumab and generic alendronate for postmenopausal osteoporotic women in Australia

**DOI:** 10.1186/s12962-016-0060-5

**Published:** 2016-10-13

**Authors:** Jonathan Karnon, Ainul Shakirah Shafie, Nneka Orji, Sofoora Kawsar Usman

**Affiliations:** School of Public Health, University of Adelaide, Adelaide, SA 5005 Australia

**Keywords:** Pharmaceutical pricing, Patent, Generic, Osteoporosis, Cost-effectiveness, Calibration

## Abstract

**Objective:**

Zoledronic acid and denosumab were funded by the Australian government for the management of osteoporosis at an equivalent price to alendronate. The price of alendronate has declined by around 65 %, but the price of the other two therapies has remained stable. Using data published since the listing, this paper reports current estimates of the value of denosumab compared to alendronate from an Australian health system perspective.

**Methods:**

A cohort-based state transition model was developed that predicted changes in bone mineral density (BMD), and calibrated fracture probabilities as a function of BMD, age and previous fracture to estimate differences in costs and QALYs gained over a 10-year time horizon.

**Results:**

The base-case incremental cost per QALY gained for denosumab versus alendronate was $246,749. There is a near zero probability that denosumab is cost-effective at a threshold value of $100,000 per QALY gained. If the price of denosumab was reduced by 50 %, the incremental cost per QALY gained falls to $50,068.

**Discussion:**

Current Australian legislation precludes price reviews when comparator therapies come off patent. The presented analysis illustrates a review process, incorporating clinical data collected since the original submission to inform a price at which denosumab would provide value for money.

## Background

Osteoporosis is a disease of the bony structure in which there is demineralization leading to a reduction in bone density, and increased risk of fracture. The most common fractures occur at the hip, spine (vertebrae) and wrist and can lead to long-lasting pain, reduced mobility, disability and sometimes death [1]. A recent burden of disease study estimated that there were 140,822 fractures due to osteoporosis and osteopenia in Australians in 2012, contributing to an annual cost of $2.75 billion, which is predicted to rise to $3.84 billion by 2022 [2].

The pharmaceutical benefits schedule (PBS) in Australia lists denosumab, alendronate, risedronate, strontium and zoledronic acid for the treatment of post-menopausal osteoporotic women. Zoledronic acid was recommended for listing in 2008 on the basis of equivalence of effect with alendronate, whilst denosumab was accepted for listing on the basis of equivalence of effect with zoledronic acid in 2010. In 2012, the Australian government introduced the expanded and accelerated price disclosure (EAPD) program that has led to significant reductions in the prices the government pays for pharmaceuticals once they come off patent [3]. Since 2010, the price of alendronate has declined by around 65 %, whilst the prices of zoledronic acid and denosumab have remained stable. In the financial year 2014/15, the government spent $120 million and $108 million on zoledronic acid and denosumab, respectively.

In the absence of new evidence to support a claim of additional effectiveness for zoledronic acid or denosumab compared to alendronate then neither therapy provides value for money when priced above alendronate. A recent meta-analysis of alendronate and denosumab concluded that denosumab does not reduce fracture risk compared to alendronate, though the four trials only reported outcomes up to 12 months [4]. Fracture risk may be improved over a longer time period due to differences in persistence to therapy. Zoledronic acid and denosumab are administered as a 6 monthly dose of subcutaneous injection, whilst alendronate is a once weekly oral regimen. Improvements in persistence as a result of the once 6 monthly dosing has been cited as a driver of increased effects for denosumab compared to alendronate [5].

Since the listing of denosumab on the PBS, an extension to the pivotal randomised placebo controlled trial of denosumab has been published [6], as well as a head-to-head comparison of persistence to alendronate and to denosumab [7]. The aim of this paper is to incorporate differences in persistence to therapy in a cost-utility analysis of an intended 5 years of treatment with denosumab compared to alendronate for post-menopausal women with osteoporosis. The analysis is reported from an Australian healthcare perspective, to assess whether denosumab (and by implication zoledronic acid) currently provides good value for money to the Australian community.

## Methods

### Cost-effectiveness model

**Fig. 1:**
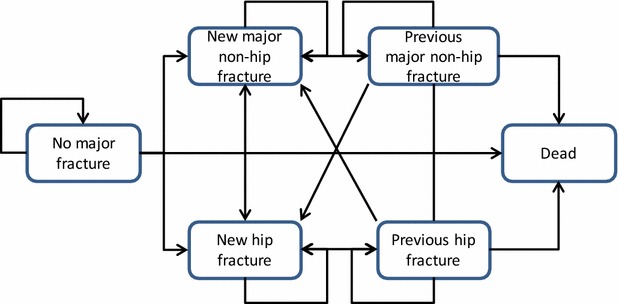
Model structure

A cohort-based state transition model was developed to represent important clinical events associated with osteoporosis. Similar to previously published models [8, 9], the model structure differentiated between hip and non-hip fractures, and between the year following a new fracture and subsequent years (Fig. 1).

The probability of fracture in each model cycle was estimated as a calibrated function of BMD, age, and the experience of prior fractures. BMD at the femoral head was represented in the model, following the use of BMD at this site in published risk equation models [10]. Annual percentage changes in BMD were estimated for women persisting with denosumab and alendronate, and for women not on treatment, over the model’s 10-year time horizon.

The eligible population was representative of the population in the fracture reduction evaluation of denosumab in osteoporosis every 6 months (FREEDOM) trial, which recruited women with mean age 72 years (range 60–90 years), mean BMD T-score at the femoral neck of −2.15, and with 24 % of women having experienced a previous fracture [11].

A 10-year time horizon was specified on the basis of the advanced age of the eligible population, and the uncertainty around long-term treatment effects and persistence. An annual model cycle with half cycle corrections was selected because the input data and the calibration data reported annual values, and the relatively small risk of fracture reduces the bias of longer model cycles [12]. The analysis represented the perspective of the healthcare provider, with costs and outcomes discounted at 5 % per annum.

### Input parameter values

Input parameter values were extracted from a range of sources, as described in Tables 1 and 2, and summarised in the following sections.

**Table 1:** Annual absolute percentage change in femoral neck BMD: observed data and scenarios for extrapolated

Year	On treatment^a^			Off treatment		
	Observed^b^	Extrapolated^c^		Observed^b^	Extrapolation scenarios^c^	
		Base case	Sensitivity scenario		Base case	Sensitivity scenario
1	2.82			0.14		
2	1.22			−0.38		
3	0.94			−0.69		
4	0.98				−0.53	−0.69
5	0.34				−0.53	−0.69
6		0.66	0.87		−0.53	−0.69
7		0.66	0.87		−0.53	−0.69
8		0.66	0.87		−0.53	−0.69
9		0.66	0.87		−0.53	−0.69
10		0.66	0.87		−0.53	−0.69

^a^Annual percentage changes in BMD assumed to be equal for patients remaining on treatment with either denosumab or alendronate

^b^Ref. [6]

^c^Base case extrapolations equal to average effects in last two years, sensitivity analyses based on average effect in last three years on treatment and in last year off treatment

**Table 2:** Model input parameter values

Parameters	Mean	Lower 95 % CI	Upper 95 % CI	Distribution	Source
*Annual non-persistence probability*					
Denosumab	0.098	0.05	0.15	Log normal	[7]
Alendronate	0.202	0.14	0.28		
*Mortality relative risk (in 1st year after a fracture)*					
Hip fracture	2.43	2.02	2.93	Log normal	[17]
Non-Hip fracture	1.65	1.52	2.17		
*Annual costs*					
Denosumab (2 × 60 mg/mL)	$541.64				PBS
Administration	$24.0				MBS
Alendronate (52 × 70 mg)	$199.16				PBS
Annual follow-up	$148.5				MBS
Hip fracture, year 1	$30,720	$24,576	$36,864	Log normal	[2]
Hip fracture, year 2+	$3280	$2624	$3936		
Non-hip fracture, year 1	$6593	$5274	$7912		
Non-hip fracture, year 2+	$253	$202	$303		
*Utility values (no fracture)*					
Mean population value (age 70–80 years)	0.8	0.78	0.82	Beta	[21]
*Utility multipliers*					
Hip fracture, year 1	0.70	0.64	0.77	Beta	[5, 22]
Hip fracture, year 2+	0.80	0.68	0.96		
Non-hip fracture, year 1	0.84	0.75	0.92		
Non-hip fracture, year 2+	0.96	0.95	0.97		
*Fracture risk equations parameters*					
a [Constant coefficient for hip fractures]	0.0005	0.0003	0.0007	Probability weights assigned (based on χ^2^ statistic)	[10], final values fitted via model calibration [13]
b [Prev. fracture coefficient for hip fractures]	1.4979	1.2659	1.7379		
c [Year coefficient for hip fractures]	0.0699	0.0367	0.1031		
d [(-BMD−1.98) coefficient for hip fractures]	0.0267	0.0159	0.0367		
e [Exponent of (-BMD−1.98) for hip fractures]	1.5042	1.2651	1.7331		
f [Constant coefficient for non-hip fractures]	0.0043	0.0022	0.0069		
g [Prev. fracture coefficient for non-hip fractures]	1.4981	1.2631	1.7374		
h [Year coefficient for non-hip fractures]	0.0698	0.0369	0.1033		
i [(-BMD−1.98) coefficient for non-hip fractures]	0.1997	0.1377	0.2704		
j [Exponent of (-BMD−1.98) for non-hip fractures]	1.4218	1.2146	1.5913		

*PBS* pharmaceutical benefits schedule, *MBS* medicare benefits schedule (drug administration assumed to require one practice nurse visit—item 10997, annual follow-up requires one GP visit—item 36, and bone densitometry procedure—item 12306)

### BMD

Table 2 presents the reported annual absolute percentage changes in femoral neck BMD for patients remaining on treatment and patients not on treatment. Parameter values for the respective groups were derived from the denosumab and placebo arms of an extension of the FREEDOM trial that reported changes in BMD over 3 years for patients not on treatment and over 5 years for patients who received denosumab for up to 5 years and who had not missed more than one dose of denosumab over the initial 3 years [6]. Beyond 3 and 5 years respectively, alternative constant annual absolute percentage changes in BMD were applied to patients not on treatment and on treatment, which were estimated from the observed data.

### Fracture risk

The model structure differentiates between the experience of hip and non-hip fractures, and represents fracture risk as a function of age, experience of previous fractures, and femoral neck BMD. Fractures of the skull, face, mandible, metacarpals, fingers, and toes, and fractures associated with severe trauma were excluded because they are not associated with decreased BMD. A published fracture risk calculator was initially used to estimate fracture probabilities [10], but the resulting model outputs did not converge to observed fracture rates in the denosumab and placebo groups in the FREEDOM extension study [6]. Instead, the following quadratic equations were fitted to predict observed hip, and non-hip clinical fracture probabilities:

$$\begin{aligned} P\left( {hip\,fracture| PF} \right) &= a(b.\,PF)\left( {1 + c.\,Year} \right) \\ \nonumber & \quad + d\left( { - BMD - 1.9} \right)^{e} \end{aligned}$$

$$\begin{aligned}P\left( {hip\,fracture| no PF} \right) &= a\left( {1 + c.\,Year} \right) \\ \nonumber & \quad + d\left( { - BMD - 1.9} \right)^{e}\end{aligned}$$

$$\begin{aligned} P\left( {non\,hip\,fracture| PF} \right) &= f(g.\,PF)\left( {1 + h.\,Year} \right) \\ \nonumber & \quad + i\left( { - BMD - 1.9} \right)^{j} \end{aligned}$$

$$\begin{aligned} P\left( {non\,hip\,fracture| no\, PF} \right) &= f\left( {1 + h.\,Year} \right) \\ \nonumber & \quad + i\left( { - BMD - 1.9} \right)^{j} \end{aligned}$$

where BMD represents the T-score at the femoral neck, Year denotes the year since treatment initiation (analogous to age), PF indicates the experience of a previous fracture, and *a* to *j* are parameter values that were fitted as part of a calibration process.

Sets of values for parameters *a* to *j* were randomly sampled from ranges specified for each of the ten input parameters. The ranges were informed by analyses of the fracture risk assessment tool (FRAX) to inform the effects of age and previous fractures on fracture risk [10], combined with deterministic model analyses to identify equation parameter values that fitted the observed fracture data. Convergence criteria were met when the model outputs for the four calibration targets (hip and non-hip fractures at 3 years, in patients receiving denosumab and placebo), were all within the 95 % confidence intervals of the observed data. Sampling continued until 5000 convergent parameter sets were identified. Probability weights were assigned to the convergent sets using the reciprocal of the χ^2^ statistic across the four calibration targets [13]. The range of convergent values for the fitted parameter *a* to *j* are presented in Table 1.

### Persistence

An intended treatment duration of 5 years is assumed for both alendronate and denosumab, but differences in persistence have been observed due to differences in the mode and frequency of administration. Persistence was defined as receiving two injections per year for denosumab [7], whilst time to non-persistence for alendronate was based on the time of a patient’s last dose [14]. Persistence on denosumab was estimated from the denosumab adherence preference satisfaction (DAPS) study, a crossover study that randomised 250 postmenopausal women to alendronate or denosumab [7]. To avoid contamination, persistence rates in the DAPS study prior to crossover were used, which were 9.5 % for denosumab and 20.2 % for alendronate. Similar non-persistence rates were reported for alendronate by a 12-month observational, multi-centre study of 6064 patients [14]. The approximately constant rate of non-persistence over 12 months supports the application of the year 1 non-persistence rates to years 2 to the maximum 5-year treatment duration.

### Treatment offset

Continuing treatment effects beyond treatment discontinuation have been analysed [15]. Extension data for alendronate show that BMD declined following treatment cessation, and that over 5 years, continuation of alendronate was associated with significantly lower risk of both non-vertebral and clinical vertebral fractures in women with lower baseline BMD or prevalent vertebral fracture [16]. The current model reflects continuing treatment effects through the application of ‘no treatment’ annual percentage changes to BMD following treatment discontinuation, which results in a gradual decline in BMD.

### Mortality

Annual probabilities for mortality were derived from Australian life tables, with adjustment for increased mortality risk in the year following a new fracture. Bliuc et al. reported standardized mortality ratios (SMRs) following hip and non-hip major fractures, reporting that the effects were maintained beyond 10 years following a hip fracture and for 5 years following a non-hip fracture [17]. The cohort-based model structure precluded the exact application of the SMRs, so both reported SMRs were applied over the full 10-year time horizon.

### Costs

Annual treatment costs for denosumab and alendronate were obtained from the Australian pharmaceutical benefits schedule: AUD $541 per year for denosumab (a twice yearly 60 mg/mL injection) and AUD $199 for alendronate (a 70 mg weekly oral drug) [www.pbs.gov.au]. To reflect the impact of patient co-payments, a sensitivity analysis was undertaken assuming all patients contributed $6.10 to each prescription (the current concession card holder contribution).

Treatment-related adverse events were not represented in the model because no statistically significant differences were observed in the main head-to-head trial of denosumab and alendronate [18]. Costs associated with the treatment of fractures, in the year following a new event, were derived from a recent Australian burden of disease study [2]. Previous studies have reported ongoing costs, beyond the year in which a fracture is experienced, based on admissions to long-term aged care facilities [19]. The reported nursing home costs in the burden of disease report (the fracture-related probability of moving into a nursing home multiplied by the annual cost) were applied as ongoing costs beyond the year in which a fracture was experienced [2]. In the absence of empirical estimates of uncertainty around the cost estimates presented in the burden of disease study, 95 % confidence limits were arbitrarily assumed to be ±25 % of each mean value in line with other studies that have defined arbitrary levels of uncertainty [20].

### Health-related quality of life (utility) weights

Age-specific general population utility weights informed a mean utility weight of 0.8 over the 10-year time horizon for women not experiencing a fracture [21]. No Australian utility weights were identified for the fracture states, and so utility multipliers reported in a recent meta-analysis were used [22], supplemented with year 2 onwards multipliers for vertebral, wrist, and other fractures estimated by Chau and colleagues [5]. A weighted non-hip fracture multiplier was estimated by applying weights to the clinical vertebral, and wrist and other fracture multipliers based on the proportions of each type of fractures reported in the FREEDOM trial [11].

### Analysis

The reference case analysis was informed by 5000 model runs, each informed by a set of input parameter values that were randomly sampled from the defined probability distributions, and the weighted sets of calibrated input parameters for the fracture risk equations [13]. The reference case outputs were analysed to generate mean estimates of costs and QALYs for denosumab and alendronate, the expected incremental cost-effectiveness ratio (ICER), as well as a cost-effectiveness acceptability curve. A comprehensive range of deterministic sensitivity analyses were also undertaken, testing the individual effects of key clinical, cost, and utility input parameters, as well as the effects of discounting and variations in price for denosumab.

## Results

### Calibration analysis

**Fig. 2:**
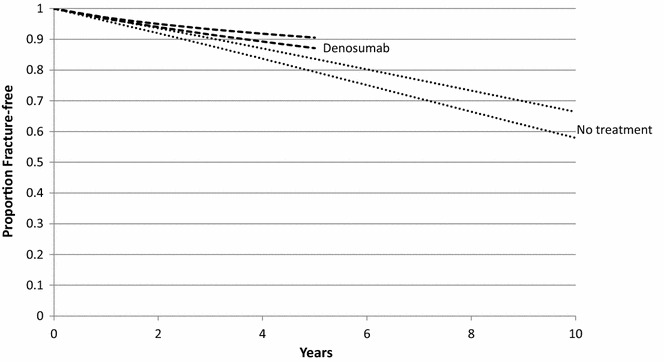
Calibrated fracture-free survival curves *upper* and *lower* bounds

Five thousand sets of convergent input parameter values were sampled from the specified ranges, each set predicted model outputs within the 95 % confidence intervals for hip, and non-hip fracture probabilities at 3 years for patients receiving denosumab, and placebo in the FREEDOM trial [6]. The upper and lower bounds of the calibrated fracture-free survival curves are presented in Fig. 2, which show 10-year hip and non-hip probabilities of 0.34–0.42 for no treatment, and 5 year probabilities of 0.1–0.13 for patients remaining on denosumab for 5 years. Increasing risk over time in the no treatment group is consistent with observational data that indicates risk increases with age in the non-treated population, whilst the trend towards decreasing risks with time for patients remaining on denosumab is consistent with the extension phase of the FREEDOM trial, which shows reducing fracture risk in years 4 and 5, as BMD continues to increase.

### Cost-effectiveness analysis

In the base case analysis, the model predicted that 24.7 and 28.5 new fractures would be experienced per 100 patients allocated to denosumab and alendronate, respectively.

Table 3 presents the cost-effectiveness results from the base case analysis, as well as from a comprehensive set of deterministic sensitivity analyses. In the base case analysis, the mean difference in cost was $1446, which is around $300 less than the mean difference in intervention costs due to lower fracture costs in the denosumab group. The reduced fracture probabilities also provide a gain of 0.006 QALYs per patient, which results in an expected ICER of $246,749 per QALY gained.

**Table 3:** Results table

Analysis	Denosumab		Alendronate		Differences		ICER
	Costs	QALYs	Costs	QALYs	Costs	QALYs	
Base case	$5483	5.823	$4037	5.817	$1446	0.006	$246,749
*Risk fracture equation parameters:*							
Lower 95 % confidence limit ICER	$5233	5.829	$3824	5.822	$1346	0.005	$191,164
Upper 95 % confidence limit ICER	$5660	5.840	$4287	5.834	$1431	0.007	$282,342
% change in BMD sensitivity scenarios (see Table 1)	$5534	5.812	$4123	5.805	$1411	0.006	$223,458
Year 3, 50 % of non-persistence in year 2	$5485	5.825	$4039	5.820	$1445	0.005	$272,323
Lower 95 % interval fracture SMR	$5498	5.860	$4107	5.855	$1391	0.006	$243,293
Upper 95 % interval fracture SMR	$5381	5.769	$3969	5.761	$1412	0.007	$197,015
Fracture costs × 0.75	$4943	5.828	$3494	5.822	$1449	0.006	$251,018
Fracture costs × 1.25	$5924	5.841	$4578	5.835	$1347	0.006	$217,746
Lower 95 % interval fracture utility multipliers	$5462	5.801	$4077	5.792	$1385	0.008	$163,384
Upper 95 % interval fracture utility multipliers	$5464	5.866	$4073	5.862	$1392	0.004	$378,871
‘No fracture’ utility = 1	$5447	7.292	$4056	7.284	$1390	0.007	$188,997
No discounting	$6403	7.117	$4913	7.109	$1490	0.008	$181,755
All patients contribute $6.10 per prescription	$5326	5.839	$3829	5.833	$1497	0.006	$260,582
Denosumab cost reduced by 50 %	$4370	5.835	$4060	5.829	$310	0.006	$50,068

*SMR* standardized mortality ratio

The deterministic sensitivity analyses show that the mean result is relatively stable to variation in most of the input parameters tested. The less conservative equation parameters and the lower 95 % confidence intervals for the fracture SMR reduce the ICER to just under $200,000. In the absence of significant survival effects, the results are heavily influenced by the utility weights, with the ICER decreasing to $163,384 when the lower interval utility weights are applied to the fracture states. Reducing the price of denosumab by 50 % reduces the ICER to $50,068.

**Fig. 3:**
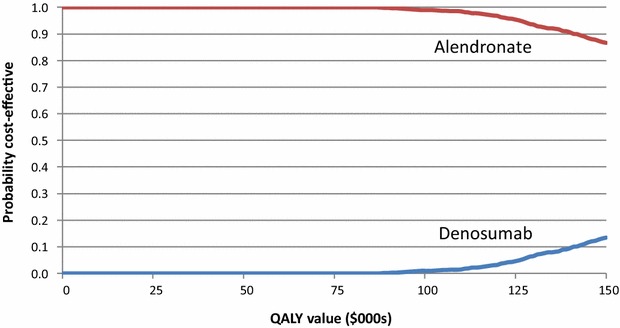
Cost-effectiveness acceptability curves

Figure 3 presents the cost-effectiveness acceptability curves, which show that at cost-effectiveness threshold of $100,000, there is a close to zero probability of denosumab being cost-effective.

## Discussion

This paper has presented an economic evaluation of denosumab compared to alendronate to re-assess the value of denosumab to the Australian community, which was evaluated for listing on the PBS in 2010. The analysis used data that has been published since 2010 to inform key model parameters and incorporates the significant price differential between denosumab and alendronate that has developed since alendronate went off-patent and was subject to the effects of the EAPD program.

Although there is some evidence of larger improvements in BMD for patients receiving denosumab compared to alendronate, it appears to be a short-term effect [18]. The pharmaceutical benefits advisory committee (PBAC) have not previously accepted claims of superior clinical effect for patients receiving either zoledronic acid or denosumab compared to alendronate and in the absence of new clinical effectiveness data there is no reason to suppose that decision would change. However, improved persistence with denosumab and zoledronic acid implies that the effective comparator for both therapies is a mix of alendronate (for the proportion of patents who persist with alendronate) and no treatment (for the proportion of patients who persist with denosumab or zoledronic acid but who would not persist with alendronate).

The base case results show that denosumab is unlikely to be considered cost-effective, with a mean ICER of $246,749 per QALY gained. Other than the price of denosumab, deterministic sensitivity analyses showed that the results were most sensitive to the assumed utility effects of fracture. The probabilistic sensitivity analysis shows a near zero probability of cost-effectiveness at a threshold value of $100,000 per QALY gained. Reducing the price of denosumab by 50 % brought the ICER down to around $50,000. PBAC has not explicitly defined a threshold for acceptable ICERs, but recent public summary documents suggest that ICERs are accepted if they are below or at the lower end of the range $45,000–$75,000 per QALY gained [23, 24]. The opportunity costs of therapies in clinical areas such as osteoporosis are likely to be greater than the average due to the large budget impact of more expensive therapies, which suggests the price of denosumb should be reduced by more than 50 % in order to demonstrate value for money to the Australian community.

Five published evaluations of denosumab compared to alendronate were identified [5, 8, 9, 19, 25], which all reported significantly lower ICERs than the current analysis, ranging from approximately Aus$30,000 [8] to around Aus$100,000 [19]. The models differed with respect to intervention prices, baseline fracture risks, SMRs, costs and utility values following fracture, discount rates and time horizons, but the main factor driving the order of magnitude difference in ICERs compared to the current study was an assumption of superior clinical effectiveness for denosumab. All five studies referred to the same meta-analysis to estimate relative risks (RRs) for alendronate compared to placebo, whilst RRs for denosumab were all derived from the FREEDOM study [11]. This non-adjusted indirect comparison provides a low level of evidence of a superior clinical effect of denosumab compared to alendronate, which was rejected by the PBAC.

In addition to the assumption of no superior clinical effectiveness, the model structure used in the current study differs significantly from the common approach used in the five reviewed evaluations [5, 8, 9, 19, 25]. The previous models all applied RRs to baseline fracture risk probabilities. An advantage of this direct application of fracture treatment effects is that it enables the use of fracture data relevant to the population for whom a funding decision is being informed by the evaluation. However, it requires an assumption of constant treatment effects and subjective assumptions regarding carryover effects after cessation of treatment.

In the current model, hip and non-hip fracture risks were calibrated separately as non-linear functions of age, BMD and previous fractures. The fitted age, BMD and previous fracture parameters reflect underlying changes in fracture risk as well as treatment effects. Whilst BMD is no longer considered a strong surrogate for fracture risk [26], the multivariate, non-linear calibration to observed BMD values and fracture event rates in treated and non-treated cohorts uses BMD as a proxy for an underlying treatment effect. The calibration process reduces the subjectivity of model assumptions around a constant treatment effect and the treatment offset period.

The analysis was based on the FREEDOM trial population [11], which reflects the PBS-listed indication for denosumab and alendronate. The mean age of the FREEDOM population was 70 years, which is similar to the mean age of the Australian population with diagnosed osteoporosis [27]. The fracture rates observed in the trial are unlikely to differ significantly from an equivalent Australian population, and the base case result was not sensitive to varying fracture risks.

The 10-year time horizon may omit some important differences in costs and outcomes between the comparators, as 75 % of the cohort remain alive at 10 years. However, given the modelled 5-year treatment duration, a 10-year horizon was selected on the basis that any residual treatment effect is likely to have dissipated after 5 years without treatment. A longer time horizon, and some adjustments to the model would be required to estimate the effects of continuing treatment beyond 5 years for patients remaining at elevated risk of fracture. The persistence input parameters were informed by survival plots over 12 months, but these parameters could be further informed by analyses of individual level PBS data to estimate real-world persistence over multiple years.

This paper has illustrated the effects of price differentials that emerge over time between pharmaceuticals that are listed on the PBS on a cost minimization basis and their comparator therapies. Recent analyses of PBS data suggest that savings of over $500 million per year could be realized if the government stopped paying differential prices for pharmaceuticals with equivalent effects [3]. Price differentials arise as comparator pharmaceuticals come off patent and are subject to price competition, whilst pharmaceuticals that remain on patent are protected from price competition. There are legislative and related political barriers to addressing this issue. Legislation was introduced in 2007 that protects the price of pharmaceuticals when their comparator goes off patent by effectively blocking reference pricing between patented and non-patented pharmaceuticals [28]. International trade agreements, with background influence from the patented-pharmaceutical industry in Australia and overseas illustrate the more general political hurdles that need to be overcome.

From a practical perspective, a review date could be specified for listed pharmaceuticals as their comparator comes off patent, to update decisions on the basis of the lower comparator cost as well as any new clinical and economic data. The analysis presented in this paper illustrates a case in which a continuing but reduced price premium may be justified on the basis of an updated cost-effectiveness analysis using data published since the original submission. In most cases, expedited reviews are likely to be sufficient to demonstrate that there is no basis for a price premium.

## Conclusions

Zoledronic acid and denosumab were funded by the Australian government for the management of osteoporosis at an equivalent price to alendronate. The price of alendronate has declined by around 65 %, but the price of the other two therapies has remained stable. This paper has reviewed the cost-effectiveness of denosumab using data published since the listing to show that the current price of denosumab (and by implication zoledronic acid) should be reduced by more than 50 % to demonstrate value to Australian community. Current Australian legislation precludes price reviews once a therapy is listed on the PBS (unless requested by a manufacturer), which means many listed pharmaceuticals are no longer providing value for money. The Australian government should act to enable a price review process to further improve the efficiency and sustainability of the PBS.

## Abbreviations

BMD: bone mineral density
DAPS: denosumab adherence preference satisfaction
EAPD: expanded and accelerated price disclosure
FRAX: fracture risk assessment tool
FREEDOM: fracture reduction evaluation of denosumab in osteoporosis every 6 months
ICER: incremental cost-effectiveness ratio
PBAC: pharmaceutical benefits advisory committee
PBS: pharmaceutical benefits schedule
QALY: quality adjusted life year
RR: relative risk
SMR: standardized mortality ratio
